# HLA Preferences for Conserved Epitopes: A Potential Mechanism for Hepatitis C Clearance

**DOI:** 10.3389/fimmu.2015.00552

**Published:** 2015-10-29

**Authors:** Xiangyu Rao, Ilka Hoof, Debbie van Baarle, Can Keşmir, Johannes Textor

**Affiliations:** ^1^Theoretical Biology and Bioinformatics, Utrecht University, Utrecht, Netherlands; ^2^Laboratory of Translational Immunology, Department of Immunology, University Medical Center Utrecht, Utrecht, Netherlands

**Keywords:** hepatitis C virus, sequence conservation, HLA disease association, peptide-MHC binding predictions, CTL responses

## Abstract

Hepatitis C virus (HCV) infections affect more than 170 million people worldwide. Most of these individuals are chronically infected, but some clear the infection rapidly. Host factors seem to play a key role in HCV clearance, among them are the human leukocyte antigen (HLA) class I molecules. Certain HLA molecules, e.g., B*27 and B*57, are associated with viral clearance. To identify potential mechanisms for these associations, we assess epitope distribution differences between HLA molecules using experimentally verified and *in silico* predicted HCV epitopes. Specifically, we show that the NS5B protein harbors the largest fraction of conserved regions among all HCV proteins. Such conserved regions could be good targets for cytotoxic T-cell (CTL) responses. We find that the protective HLA-B*27 molecule preferentially presents cytotoxic T-cell (CTL) epitopes from NS5B and, in general, presents the most strongly conserved epitopes among the 23 HLA molecules analyzed. In contrast, HLA molecules known to be associated with HCV persistence do not have similar preferences and appear to target the variable P7 protein. Overall, our analysis suggests that by targeting highly constrained – and thereby conserved – regions of HCV, the protective HLA molecule HLA-B*27 reduces the ability of HCV to escape the cytotoxic T-cell response of the host. For visualizing the distribution of both experimentally verified and predicted epitopes across the HCV genome, we created the HCV epitope browser, which is available at theory.bio.uu.nl/ucqi/hcv.

## 1 Introduction

Hepatitis C virus (HCV) is a severe infectious agent and affects millions of people per year (1). Approximately 25% of the infected individuals spontaneously resolve the infection, whereas most develop a persistent viral infection, which may lead to liver cirrhosis, liver failure, or hepatocellular carcinoma (2). Although the determinants of HCV clearance, persistence, and disease outcome are not well known, it has been shown in numerous studies that the magnitude, diversity, and quality of T-cell responses play an essential role in controlling the infection and determining the outcome of HCV infection (3–6). Especially, efficient (CD4^+^) T-cell responses against non-structural proteins might be important for viral control (7, 8). The importance of T-cell responses is also supported by the finding that certain human leukocyte antigen (HLA) molecules are associated with differential HCV infection outcome. Numerous HCV-HLA association studies have been performed covering different ethnic groups and different HCV genotypes [reviewed in Ref. (9, 10)]. Interestingly, two allele groups, B*27 and B*57, which have previously been associated with slow disease progression in HIV-1-positive individuals [reviewed by Bashirova et al. (11)], are also associated with viral clearance in HCV infection. For HLA molecules in these groups, which we will call *protective alleles*, several dominantly targeted CTL epitopes have been identified. These epitopes were shown to contain mutations in individuals unable to clear the HCV infection (12, 13) and lead to fitness loss (14, 15) unless restored by compensatory mutations.

The HCV genome encodes a polyprotein precursor of about 3000 amino acid residues, which is processed into several mature structural proteins (Core, E1, E2) and non-structural proteins (NS2, NS3, NS4A, NS4B, NS5A, NS5B, P7) (16). A possible mechanism behind the observed associations between certain HLA class I molecules and HCV infection outcome could be the differential presentation of HCV proteins by certain HLA molecules, as was shown to be the case for HLA associations with HIV-1 (17) and HTLV-1 (18). Such protein targeting could be beneficial for various reasons. For instance, the targeted proteins could be highly expressed, or the timing of their expression could exhibit a critical period which is essential for viral replication. Alternatively, a targeted protein could harbor many conserved epitopes in which mutations would come at a high fitness cost, making it more difficult to escape from the CTL response. Here, we therefore address two related questions: (1) Is there evidence for differential presentation of HCV proteins by protective alleles? and (2) Do protective alleles target conserved regions of the HCV genome?

To address these questions, we analyzed the HCV epitope repertoires of several HLA molecules that have been associated with viral clearance or persistence, employing experimental data from public databases and *in silico* predictions of HLA-peptide binding to define these HLA epitope repertoires. We found that the protective HLA molecule B*27 shows a preference to present epitopes from the HCV protein NS5B, whereas other HLA molecules show no preferential targeting or target other HCV proteins such as P7. Analyzing the sequence variability of HCV proteins, we found that NS5B harbors the largest fraction of strongly conserved regions among all HCV proteins and that the predicted B*27 epitope repertoire contains the largest amount of strongly conserved epitopes of all alleles that were analyzed. Taken together, our analysis suggests a relationship between the protective potential of an HLA molecule and the degree of sequence conservation of the HCV epitopes targeted by that HLA molecule.

## 2 Materials and Methods

### 2.1 Experimentally Verified HCV T-Cell Epitopes

**Figure 1:**
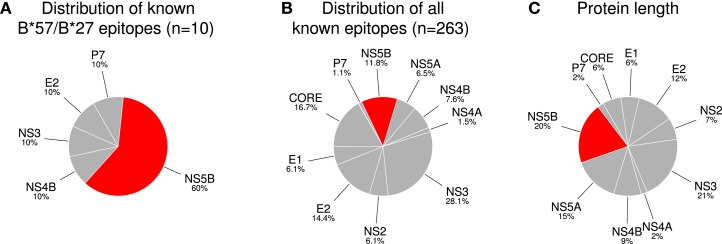
NS5B is enriched in experimentally verified epitopes restricted by protective HLA allele groups (HLA-B*27, HLA-B*57). **(A)** The distribution of (non-redundant) experimentally verified epitopes restricted by the protective alleles. **(B)** The distribution of all non-redundant HCV epitopes reported in the IEDB and LANL databases. **(C)** The normalized length of each protein in the complete HCV proteome. The abundance of NS5B in **(A)** significantly differs from what would be expected based on the distributions in **(B)** (p = 0.0006) and **(C)** (p = 0.006; Fisher’s exact test).

All experimentally verified HCV CD8^+^ T-cell epitopes restricted by HLA class I molecules were downloaded (October 2014) from two public databases: (1) the Los Alamos HCV immunology database^1^ [Ref. (19); note that maintenance of this database stopped in 2007] and (2) the Immune Epitope Database and Analysis Resource^2^ (IEDB (20)). HCV is classified into 7 phylogenetically distinct genotypes (21). As HCV genotype 1 is the most dominant strain worldwide and is well studied (22), we aligned each known epitope to the HCV reference strain H77 (accession number AF009606) using the blastp program (23). Only those epitopes for which an alignment with 100% source coverage could be found were included in the analysis. To make sure that all epitopes we considered are from the HCV strains infecting humans, epitopes identified using HLA transgenic mammalian cells were excluded. This procedure resulted in 398 experimentally verified combinations of an HCV genotype 1 CTL epitope and its known HLA restriction (26 peptides appear in more than one combination). Only 7 epitopes were restricted by HLA-C molecules. To calculate the distribution of CTL epitopes over the HCV proteins for each HLA allele, we limited our analysis to 263 non-redundant experimental epitopes: whenever multiple epitopes aligned to the same positions in the H77 reference strain, we only included the epitope with the highest alignment score. The final set of curated epitopes, which forms the basis for Figure 1, is provided as supplementary data (Data Sheet S1 in Supplementary Material).

### 2.2 HLA-Peptide-Binding Predictions

We used the artificial neural network-based MHC binding predictor NetMHCpan 2.8 (24) to predict MHC binding affinities for 9-mer peptides from the HCV reference strain H77 (accession number AF009606). For every allele group, 2-digit resolution, we have performed the predictions for the most dominant allele, 4-digit resolution. Alternatively, we predicted MHC binding for common HLA-A and HLA-B alleles by using the Stabilized Matrix Method (25), but we found that this did not affect the conclusions drawn from our analysis so we omit these data from the present article for the sake of simplicity. We also performed a version of our analysis in which we additionally used the algorithm NetChop (26) to predict proteasomal cleavage and transportation by TAP, which are important selection steps during peptide generation for antigen presentation. Also this analysis gave results similar to those reported here.

### 2.3 Sequence Conservation

Prealigned HCV protein sequences (Core, E1, E2, NS2, NS3, NS4A, NS4B, NS5A, NS5B, and P7 sequences) dated 2008 or older were downloaded (October 2014) from the Los Alamos HCV sequence database. The alignments contain only the “oldest” sequence per patient based on their GenBank submission date and cover sequences from all HCV genotypes. To estimate sequence conservation, the Shannon entropy

$$E\left(i\right)=-\underset{L}{\sum }p_{L}\log_{2}\left(p_{L}\right)$$

was calculated for each position *i* of each protein, where *p_L_* is the observed frequency of a certain amino acid *L* at position *i* in the alignment. The Shannon entropy for position *i* reaches its maximum value of log_2_(20) (≈4.3) if all amino acids are equally frequent at that position and is 0 if the position is fully conserved. Different numbers of sequences were available for different HCV proteins, ranging from 466 sequences for NS5B to 918 sequences for NS3. To prevent a bias due to different numbers of sequences in the alignments, we used the Miller-Madow entropy estimator which corrects for such bias (27). We repeated this analysis separately for sequences of HCV genotype 1.

To map sequence conservation of the HCV genome at the spatial resolution that is most relevant for epitope presentation, we applied a running median filter with window size nine to obtain an estimate of the overall conservation of a nine-mer centered at the respective position. The window size nine was chosen because it matches the typical length of peptides presented by HLA class I molecules. The first and last four positions of each protein were not assigned a median entropy, as no nine-mer is centered at these positions. We chose the median filter instead of other alternatives such as a running average because the distribution of entropies is strongly skewed. In order to separate strongly conserved regions from more variable regions, we applied a threshold of 0.037 bits – the lower quartile of the overall entropy distribution in the HCV proteome. Changing this threshold did not affect the qualitative results.

### 2.4 Statistical Analysis

All statistical analysis was performed using the R environment (28). To analyze the targeting of proteins by HLA alleles, we ranked the 9-mer peptides from each protein by their predicted binding affinities and considered the *n* best-ranking peptides as epitopes, where *n* was varied as described in the results. We then compared the resulting distribution of epitopes across proteins to the distribution expected from a random ordering of the peptides. We used a chi-square test to investigate the significance of the difference between the expected and observed distributions and computed standardized residuals for each HLA-protein combination to assess the sources of those differences.

## 3 Results

### 3.1 HCV-HLA Associations and Analysis of Known HCV Epitopes

We studied the HLA restriction of all known CTL epitopes from the HCV LANL and IEDB databases (see Methods). Kuniholm et al. (9) conducted a literature review of HCV-HLA associations. Using stringent selection criteria, the authors identified five HLA class I allele groups (B^⋆^18, B^⋆^27, B^⋆^57, Cw^⋆^01, and Cw^⋆^04) that are strongly associated with either HCV clearance or persistence (Table 1). Interestingly, the two allele groups B^⋆^27 and B^⋆^57, which are strongly associated with slow disease progression in HIV-1-positive individuals, are also associated with a favorable infection outcome in HCV-infected patient cohorts. Next, Kuniholm et al. (9) tested the association of HLA alleles with HCV viremia in a large cohort of HCV-seropositive women and confirmed all the associations, except for B^⋆^18, though the effects were not always significant. Of the list of protective HLA allele groups, multiple CTL epitopes have only been reported for B^⋆^27 and B^⋆^57 (Table 2). We observed that 6 out of the 10 experimentally verified HCV CD8^+^ T-cell epitopes restricted by the protective HLA allele groups are located in the HCV protein NS5B, whereas the remaining 4 epitopes are distributed over 4 other HCV proteins (Figure 1A). The share of NS5B epitopes from B^⋆^57 and B^⋆^27 is larger than would be expected from the distribution of all known epitopes across HCV proteins (Figure 1B) or the protein length (Figure 1C). Thus, this small set of experimentally verified epitopes suggests that the epitopes of protective alleles are concentrated in the NS5B protein.

**Table 1:** **HLA class I allele groups with significant associations with HCV viremia as reported by Kuniholm et al. (9)**.

Allelegroup^a^	HCV RNA status	Reference	Population	N_ep_^b^
B*18	Persistence	(29)	Irish	1
B*27	Clearance	(29)	Irish	6
B*57	Clearance	(9, 12, 30, 31)	Mixed	4
Cw*01	Clearance	(9, 29, 30)	Caucasian	0
Cw*04	Persistence	(30, 32)	Caucasian, Irish	0

*^a^Two-digit resolution*.

*^b^Number of genotype 1 CTL epitopes identified so far (counting only non-redundant epitopes; see Materials and Methods)*.

**Table 2:** **Experimentally verified CTL epitopes restricted by protective HLA alleles (used in Figure 1A)**.

Epitope	Protein	Position^a^	HLA	Predicted IC50 (nM)^b^
***GRGKPGIYR***F	NS3	466–475	HLA-B*27	1195
KGGRKP***ARLIVFPDL***	NS5B	151–165	HLA-B*27	484
ARHTPVNSW	NS5B	400–408	HLA-B*27	1447
ARMILMTHF	NS5B	421–429	HLA-B*27	129
GRAAICGKY	NS5B	516–524	HLA-B*27	122
GRWVPGAAY	P7	34–42	HLA-B*27	100
***NTRPPLGNW***F	E2	158–167	HLA-B*57	241
LTTSQTLLF	NS4B	90–98	HLA-B*57	306
KSKKTPMGF	NS5B	209–217	HLA-B*57	121
***LGVPPLRAW***R	NS5B	492–501	HLA-B*57	157

*^a^Position with respect to the HCV reference strain H77*.

*^b^For peptides longer than 9 amino acids, the nine-mer with the best binding affinity is shown in bold italics, and this is the binding affinity reported in the last column*.

### 3.2 Preferential Targeting Patterns of HCV Proteins by Class I HLA Molecules

The power of our analysis of experimental epitopes is limited because for many alleles, no CTL epitopes have been reported yet. We therefore used *in silico* binding prediction as an alternative approach to define HLA epitope repertoires (see Materials and Methods for details). The cohort used by Kuniholm et al. (9) to define protective allele groups (Table 1) was largely infected with HCV genotype 1 (55% with genotype 1a, 29% with genotype 1b, and 4% with genotype 1 of undetermined subtype). Therefore, we assumed that the associations identified by Kuniholm et al. (9) should be valid in the context of HCV genotype 1 infection and used the genotype 1 reference strain H77 from the LANL HCV database (19) for our predictions. The HLA-binding affinities of all HCV peptides of length nine were estimated for several HLA alleles using the prediction tool NetMHCpan (see Materials and Methods). This method predicts an IC50 value of <500 nM for 8 out of the 10 known HLA-B^⋆^27 and HLA-B^⋆^57 epitopes that are part of Figure 1A (Table 2). Therefore, the *in silico* predictions should be a valid approach to overcome the limited number of experimentally identified epitopes.

**Figure 2:**
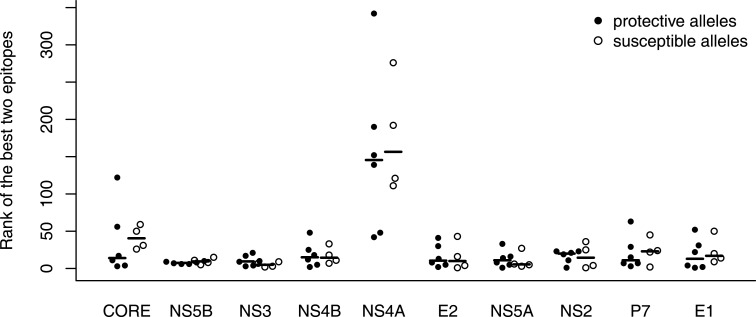
**Binding affinity rankings of the best epitopes do not differ between susceptible and protective alleles**. The relative ranks of epitopes from each HCV protein are plotted for the protective (B*5701, B*2705, and Cw*0101; filled circles) and susceptible (B*1801, Cw*0401; open circles) HLA alleles (see Table 1). The median of each group is indicated by a horizontal line. The relative rank is calculated with respect to the binding scores of all peptides from the HCV proteome and separately for each HLA allele. None of the differences between susceptible and protective alleles are significant at α = 0.05.

In a first attempt to study preferential targeting of HCV proteins by protective and susceptible HLA alleles, we followed the strategy taken in previous studies on HIV-1 (17) and HTLV-1 infection (18). We ranked the predicted HCV epitopes by their predicted HLA-binding affinity within all HCV proteins and then determined the ranks of the two best-binding peptides derived from each HCV protein among all other HCV epitopes (Figure 2). These ranks are expected to depend on the protein length (Figure 1C): shorter proteins are less likely to contain many top-ranking peptides than long proteins. This intuition was confirmed for the NS4A protein, which is the shortest of the HCV proteins (56 amino acids). Interestingly, the ranks for the second shortest protein P7 (64 amino acids) were much higher and comparable to the other proteins. However, this analysis did not show any convincing differences between protective and susceptible alleles (Figure 2).

We reasoned that focusing only on the top two binders might not be an optimal approach because of the low number of peptides involved, which is expected to produce significant uncertainty with respect to the ranking. Thus, we decided to alternatively look at the whole set of peptides that are predicted to bind each HLA class I molecule. The fraction of peptides bound by each HLA is normally considered to be around 2%. We therefore looked at the top 30–120 binders (corresponding to 1–4% of the HCV proteome, respectively) and compared the observed distribution of proteins among those binders to the expected distribution based on the protein length. Overall, HLAs seem to deviate significantly from uniform protein targeting (p = 0.007, chi-square test, for the 120 binders cutoff). To investigate which HLA-protein combinations gave rise to the difference between the observed protein targeting and a uniform targeting based only on protein length, we investigated the standardized residuals of the chi-square test. Standardized residuals measure the difference between an observed frequency and the expected frequency; a positive residual means that a combination occurs more often than expected, whereas a negative residual means that it occurs less often than expected. The magnitude of the residual gives an indication of statistical significance. For instance, a standardized residual of 2 or −2 means that the difference is twice as large as the standard error in measuring the expected frequency and roughly corresponds to a significance level of α = 0.05. In the following, we will call the residuals *enrichment scores*.

**Figure 3:**
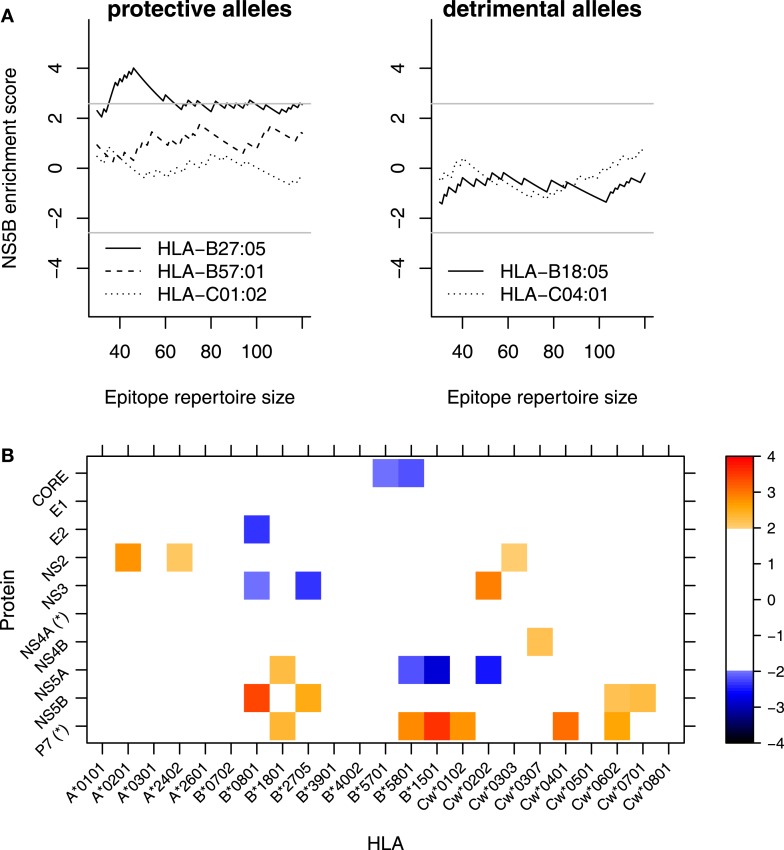
HLA-B*27 targets the NS5B protein. **(A)** Enrichment score (standardized residual; explained in main text and Materials and Methods) as a function of the rank cutoff between epitopes and non-epitopes. For example, at a cutoff of 60, the epitope repertoire consists of the 60 best binders and the proportion of predicted epitopes from a given protein is compared with the proportion that would be expected based on the protein length. An enrichment score of 2 roughly corresponds to significance at α = 0.05 and a score of 2.58 corresponds to α = 0.01 (gray horizontal line). The top 60 binders correspond to the top 2% binders in HCV. **(B)** Matrix plot of enrichment scores per protein-HLA combination. Enrichment scores in each cell are averaged over cutoffs between 60 and 120 (or the top 2–4%). For clarity, all cells that would not be individually significant at the α = 0.05 are muted (white cells). *These proteins are shorter than 100 amino acids (aa). Therefore, the enrichment scores are based on only a few peptides. The protein lengths are CORE 191aa; E1 192aa; E2 363aa; P7 63aa; NS2 217aa; NS3 631aa; NS4A 54aa; NS4B 261aa; NS5A 448aa; and NS5B 591aa.

In contrast to the previous analysis (Figure 2), the enrichment scores revealed differential targeting for many HLA molecules. Specifically, plotting the scores as a function of the cutoff shows that HLA-B^⋆^2705, but none of the other protective or susceptible HLA alleles, targets NS5B (Figure 3A). We also performed a two-dimensional analysis of the targeting patterns of all HLA-protein combinations by averaging the standardized residuals across the cutoffs from 60 to 120 (Figure 3B), which showed a rich pattern of protein targeting by many HLA alleles. Including a filter of the predicted HLA ligands for their probability to be generated by the proteasome (26) does not appreciably change these results (data not shown).

Taken together, both the experimentally determined and the predicted epitope repertoires indicate that the protective HLA-B^⋆^27:05 allele targets the NS5B protein of HCV.

### 3.3 Identifying Strongly Conserved Regions of the HCV Genome

Targeting NS5B could be important for several reasons. For example, NS5B could be expressed in infected cells in large amounts or early during the infection, resulting in both cases in an efficient antigen presentation and a good CTL response. Unfortunately, little is known about the expression of HCV proteins in infected cells. Alternatively, since HCV is known to escape CTL pressure by continuous evolution (33), CTL responses to structurally and functionally constrained parts of the HCV genome are likely to be most beneficial. Thus, a possible mechanism how targeting of NS5B by HLA-B^⋆^27 would increase the odds of viral clearance could be that NS5B harbors many constrained epitopes. Therefore, we sought to identify conserved regions in the HCV genome and to compare their locations with those of HLA-B^⋆^27 epitopes.

**Figure 4:**
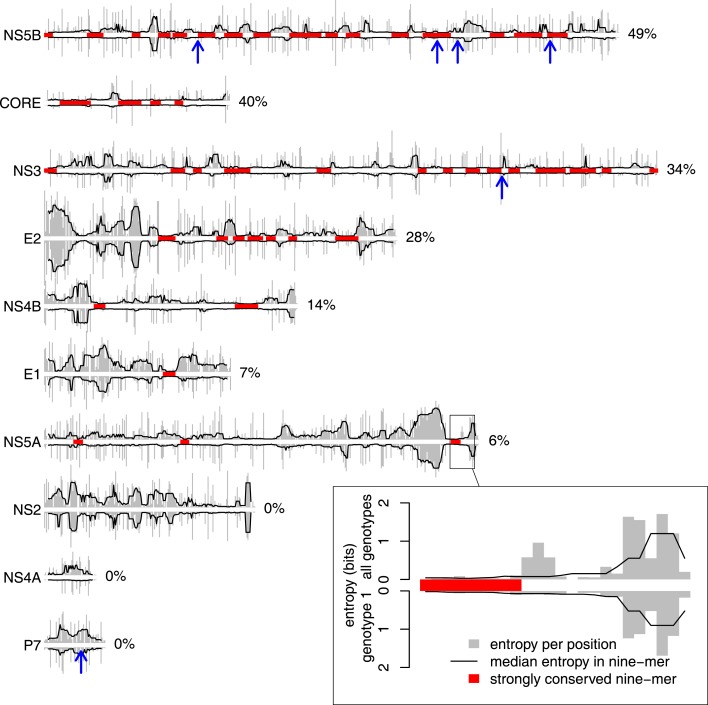
**NS5B is the most conserved HCV protein**. Protein variability was estimated using bias-corrected Shannon entropy scores calculated for each position in HCV protein alignments (see Materials and Methods). For each protein, the plot shows raw entropies (gray bars) and median entropies in nine-mers (black lines) estimated from all sequences (plotted upward) or genotype 1 sequences only (plotted downward; see inset for the scale). Blue arrows point to the *centers* of experimentally verified HLA-B*27 epitopes (see Figure 1). Strongly conserved nine-mers with low median entropy (see Materials and Methods and main text for details) are highlighted in red. According to the coverage by strongly conserved nine-mers (shown to the right of each plot), NS5B is the most conserved HCV protein (49%). The number of sequences per protein varies between 466 (NS5B) and 918 (NS3).

The relative variability of HCV proteins was assessed by estimating the average Shannon entropy of all positions for each protein using prealigned amino acid sequences from *all* genotypes available from the Los Alamos HCV database. Each of these alignments includes only one sequence per patient. In order to allow for direct comparison between the proteins, we corrected for the number of sequences per protein alignment (see Materials and Methods). We then identified strongly conserved regions in the HCV genome as nine-mers in which the median entropy was lower than the lower quartile of the overall entropy distribution; this was the case for about 10% of all nine-mers in the genome. Not surprisingly, strongly conserved regions are not uniformly distributed across HCV proteins (Figure 4). Almost half of the sequence of NS5B and Core are covered by strongly conserved regions, whereas such regions are not found at all in the NS2, NS4A, and P7 proteins. The degree of conservation probably reflects the structural flexibility of these proteins: the structural protein Core, for example, constitutes the capsid of the virus particle and affects several cellular processes (34). For most of the HCV proteins, however, the functional and structural constraints are not yet known. The number of sequences available is not evenly distributed over the different HCV genotypes. Therefore, we could only repeat the conservation analysis on a genotype basis for genotype 1, as the number of available sequences per protein is less than 50 for the other genotypes. The sequence conservation within genotype 1 (plotted downward in Figure 4) largely mirrors the conservation across all genotypes (plotted upward), a notable exception being the NS4A protein which is quite conserved within genotype 1 but highly variable across all genotypes.

### 3.4 Presentation of Strongly Conserved Epitopes by HLA-B*27

We mapped the six experimentally identified HLA-B^⋆^27 epitopes to the multiple sequence alignment used for assessing variability of the HCV genome (blue arrows in Figure 4). Four of the epitopes lie within or border of strongly conserved regions of NS5B and NS3, one lies within a less conserved region of NS5B, and one is found in the short highly variable P7 protein. While these sequence conservation of these epitopes thus largely reflects the average sequence conservation of the proteins themselves, the small number of known HLA-B^⋆^27 epitopes does not allow meaningful statistical assessment.

**Figure 5:**
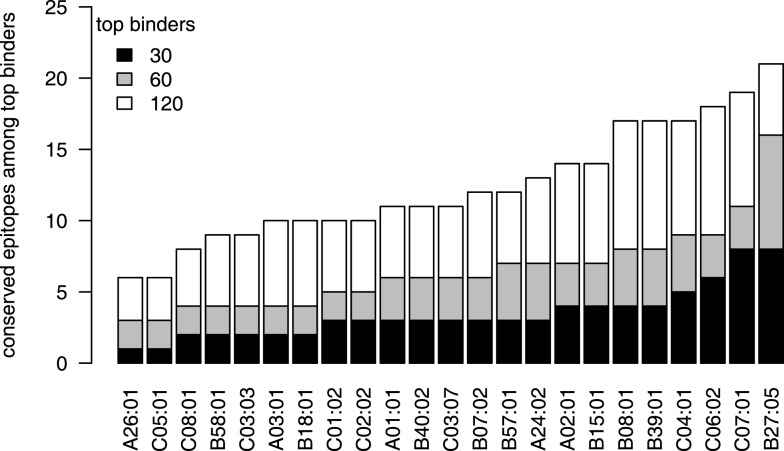
HLA-B*27 presents the most conserved HCV epitopes. For each HLA supertype, the number of strongly conserved epitopes among the top 30 (black), top 60 (gray), and top 120 (white) predicted binders is shown. HLA-B*27 shows the largest fraction of highly conserved epitopes for all three thresholds.

Therefore, we also analyzed the localization of *in silico* predicted epitopes for all HLA alleles used in Figure 3 using the top 30, 60, and 120 binders, which correspond to the top 1%, 2%, and 4%, as explained above. For all three cutoffs, HLA-B^⋆^27 is predicted to present the largest number of strongly conserved epitopes of all the HLA alleles that were analyzed (Figure 5), whereas such a preference was not found for HLA-B^⋆^57. As was the case for the few experimentally verified epitopes, the epitope conservation level of the predicted epitopes mirrors the protein targeting pattern (Figure 3): indeed, 11 of the 16 strongly conserved predicted epitopes for HLA-B27 lie within NS5B (60-binders cutoff; Table 3).

**Table 3:** Predicted HLA-B*27 epitopes that have both low entropy and high binding affinity.

Epitope	Protein	Position^a^	Entropy (bits)^b^	Predicted IC50 (nM)
RRGPRLGVR	CORE	39–47	0.03	132
SRCGSGPWI	E2	212–220	0.037	1003
YRLWHYPCT	E2	230–238	0.037	586
RRGRTGRGK	NS3	461–469	0.036	234
NRLIAFASR	NS4B	206–214	0.018	865
ERLYVGGPL	NS5B	258–266	0	786
YRRCRASGV	NS5B	276–284	0.024	491
RRCRASGVL	NS5B	277–285	0.024	115
ARAAWETAR	NS5B	393–401	0.024	156
QRLHGLSAF	NS5B	464–472	0.024	496
LRKLGVPPL	NS5B	489–497	0	438
RKLGVPPLR	NS5B	490–498	0	824
LRAWRHRAR	NS5B	497–505	0.024	251
WRHRARSVR	NS5B	500–508	0.024	238
HRARSVRAR	NS5B	502–510	0.024	264
ARSVRARLL	NS5B	504–512	0.024	170

*^a^Position with respect to the HCV reference strain H77*.

*^b^The median entropy in the nine-mer*.

Taken together, the *in silico* analysis as well as our analysis based on a limited number of known HCV CD8^+^ T-cell epitopes both suggest that the protective HLA allele HLA-B^⋆^2705 associated with clearance of HCV viremia tends to target conserved regions within the NS5B protein, whereas many other HLA molecules target different (and less conserved) regions of the HCV genome. However, the *in silico* analysis did not confirm such an effect for the other protective HLA alleles.

## 4 Discussion

Several studies have shown that both CD4^+^ and CD8^+^ T-cell responses play important roles in controlling HCV replication during infection (3–6), and certain HLA alleles have been significantly associated with differential infection outcome (9). The underlying mechanism behind these associations remains unclear. In case of HIV-1 and HTLV-1, it was shown that the immune system can effectively control infectious diseases by targeting certain pathogen-derived proteins (17, 18). Similarly, other studies found that T-cell epitopes are not equally distributed over the genome of HIV-1 and *M. tuberculosis* but that they cluster together in conserved regions (35–37). We hypothesized that targeting of conserved regions within the HCV proteome by HLA alleles could be a potential mechanism to control viral replication during the acute phase of HCV infection, which could then further determine disease outcome.

To test this hypothesis, we performed a literature study to compile a list of protective and susceptible HLA alleles. This turned out to be rather difficult due to limited data and differences between ethnic groups. Using the five strong HLA class I/HCV associations defined by Kuniholm et al. (9) as a basis for our analysis, we found that experimentally verified CD8^+^ T-cell epitopes restricted by the protective HLA molecules HLA-B^⋆^27 and HLA-B^⋆^57 are enriched within the NS5B protein. When carrying out a similar analysis using *in silico* predicted epitopes, we could only see a preference for presentation of NS5B by HLA-B^⋆^2705, but not for HLA-B^⋆^57:01. Interestingly, however, for precisely this HLA-protein combination, several epitopes have been identified for which mutations appear to have a high fitness cost (15, 38). In line with previous studies (39), we found that NS5B is the most conserved protein, followed by Core and NS3, while the other proteins harbor more highly variable regions. Combined with these results, our study thus suggests that the protective effect of HLA-B^⋆^2705 might be mediated by preferential targeting of conserved epitopes.

Unexpectedly, we found that quite many HLA class I alleles seem to preferentially present epitopes from the P7 protein (Figure 3B), which according to our analysis does not harbor any conserved regions. It is interesting to observe that the HLAs targeting P7 include the two detrimental alleles B^⋆^18 and Cw^⋆^04. Given that P7 is also the protein whose functional role for HCV infection is most unclear (40), it is tempting to speculate that the P7 targeting prevents those HLAs from inducing more protective responses to other proteins. However, two caveats are that (1) the protective allele Cw^⋆^01 also targets P7 and (2) P7 is the second shortest HCV protein, hence the uncertainty involved in ranking its best epitopes is high compared to longer proteins like NS5B.

HCV genotype differences also play a role in potential protective effects. It was shown, for example, that the protective effect of B^⋆^27 is dependent on the genotype, with genotype 1-infected patients having T-cell responses against the protective B^⋆^27 epitope, and genotype 3-infected patients showing no evidence of T-cell responses to any epitope from the same region in the HCV proteome, which differed in its sequence by three amino acids (13). Our analysis focused on genotype 1 sequences (i.e., we use genotype 1a and 1b reference strains in our *in silico* analysis and analyze only experimentally verified CTL epitopes from genotype 1 sequences), as HLA associations taken into account here were defined largely in the context of HCV genotype 1 infections. When more sequence and epitope data become available from the other genotypes, it will be possible to repeat our analysis on a genotype basis.

So far, there is no direct evidence that targeting more conserved HCV proteins by CD8^+^ T cells helps controlling HCV replication. However, it has been widely argued that CD8^+^ T-cell responses specific for conserved regions of HIV-1 (especially Gag p24) should slow down disease progression (17, 38, 41). As in the case of HIV-1, the most conserved HCV proteins are also the most functionally and structurally constrained ones, which makes it more difficult for the pathogen to escape from host immune control in these proteins than in other regions. In line with this reasoning, it has been shown that a point mutation within a B^⋆^27 restricted dominant T-cell epitope in NS5B (pos. 421-429, ARMILMTHF) entails a large fitness cost (15). A study of viral evolution in non-structural proteins during the acute phase of HCV infection showed that mutations in this NS5B epitope are also found and maintained in subjects that are not B^⋆^27 positive (42). This lack of reversion over time suggests that additional (compensatory) mutations must have occurred, which do not allow simple reversion. Moreover, a rare mutation in the anchor residue of a subdominant B^⋆^27 restricted NS5B epitope (pos. 516-524, GRAAICGKY) was shown to nearly abrogate viral replication, which could be restored by two upstream mutations (38). Interestingly, also for B^⋆^57, it has been shown that mutations in an NS5B epitope (pos. 209-217, KSKKTPMGF) substantially impaired viral replication, which was restored by compensatory mutations further upstream (14). Another recent study found that B^⋆^57-positive HCV-infected chronic carriers harbor HCV strains with a high frequency of mutations in key residues of this NS5B epitope as well as in an E2 epitope [pos. 158-166, NTRPPLGNW; Ref. (12)]. These epitope variants were also shown to be recognized to a lesser extent in chronic carriers, which indicates that presentation of these epitopes is important in viral clearance. Unfortunately, to our knowledge, none of the other known B^⋆^57 or B^⋆^27 epitopes were studied in such detail. Still, these data indicate that NS5B is a highly constrained region of the HCV genome and shed some light on why immune responses targeting this conserved protein of HCV might result in a favorable infection outcome.

We wish to stress that the research presented in this article is exploratory and that more clinical data are required to test the mechanistic hypothesis suggested by our results. Specifically, the sixteen predicted epitopes shown in Table 3 should be good CTL targets, as they have low entropy and high predicted binding affinity. This prediction is testable by measuring CTL responses against these epitopes *in vivo* and correlating the strength of immune escapes against these epitopes to the ability to clear the HCV infection.

Going beyond hepatitis C infections, we believe that the methodology we developed here for measuring HLA protein targeting and epitope distributions may be useful for studying class I responses to other infections as well. To explore the epitope distributions identified in this study for both experimentally verified and *in silico* predicted epitopes, we have generated the “HCV epitope browser” – an extended, interactive version of Figure 4. This tool is available at theory.bio.uu.nl/ucqi/hcv.

## Author Contributions

CK, DB, and JT designed the research. XR, IH, and JT performed the research and analyzed the data. XR, IH, DB, JT, and CK wrote the manuscript.

## Conflict of Interest Statement

The authors declare that the research was conducted in the absence of any commercial or financial relationships that could be construed as a potential conflict of interest.

## Footnotes

^1^http://hcv.lanl.gov/content/immuno/immuno-main.html

^2^http://www.immuneepitope.org/
